# Extreme delta brush patterns guide the complex motor phenomenon of anti-NMDA receptor encephalitis: A case report

**DOI:** 10.1097/MD.0000000000019384

**Published:** 2020-02-28

**Authors:** Yu-Ming Chen, Pei-Hsin Kuo

**Affiliations:** a Department of Neurology, Hualien Tzu Chi Hospital, Buddhist Tzu Chi Medical Foundation; b Tzu Chi University, Hualien, Taiwan.

**Keywords:** anti-NMDA receptor encephalitis, extreme delta brush, movement disorder, seizure

## Abstract

**Rationale:**

Anti-N-methyl-D-aspartate receptor (anti-NMDAR) encephalitis is an autoimmune disease that is associated with cell-surface NMDAR-targeting autoantibodies. Typical anti-NMDAR encephalitis symptoms include psychosis, seizure and extrapyramidal side effects. However, early diagnosis of anti-NMDAR encephalitis remains challenging due to the complexity of the motor phenomenon.

**Patient concerns:**

Here, we report a new diagnosis of anti-NMDAR encephalitis in a young woman with a history of epilepsy.

**Diagnoses:**

Electroencephalography revealed a typical “extreme delta brush” pattern, which indicated anti-NMDAR encephalitis.

**Interventions:**

The clinical status of the patient markedly improved after immediate and aggressive immunosuppression therapies.

**Outcomes:**

The patient was discharged with mild cognitive impairment. However, this was completely resolved 1 month postdischarge.

**Lessons:**

We conclude that subacute onset focal seizure with psychosis as well as compatible electroencephalography findings (i.e., extreme delta brush patterns) should be considered notable early indicators of anti-NMDAR encephalitis. This would ensure early and effective clinical interventions, which are essential for favorable outcomes.

## 1 Introduction

Anti-N-methyl-D-aspartate receptor (anti-NMDAR) encephalitis is an autoimmune disease caused by autoantibodies that target cell-surface NMDARs. Anti-NMDAR encephalitis patients typically present with viral prodrome, followed by subacute psychiatric symptoms such as delusion, hallucination, movement abnormalities, and seizure.^[1]^ If left untreated, more severe symptoms such as dysautonomia or coma may develop. However, as anti-NMDAR encephalitis-related movement disorder is complex and often intermingled with those of other conditions such as focal seizure,^[2]^ accurate diagnosis of anti-NMDAR encephalitis remains challenging.

A previous study has reported the “extreme delta brush” (EDB), a unique electroencephalography (EEG) pattern, in NMDAR encephalitis patients.^[3]^ EDB is present in only one-third of anti-NMDAR encephalitis patients and is characterized by beta bursts overriding on delta waves in EEG analysis.^[4]^ Here, we report a new anti-NMDAR encephalitis case that was initially diagnosed by the EDB pattern observed in EEG analysis.

## 2 Case presentation

Our patient was a 20-year-old woman with a history of aseptic meningitis (6 years before) and focal epileptic sequelae (left face, left arm, and left leg muscle twitch). Seizure recurred 3 years after previous follow-up was terminated, and the patient returned to our neurology clinic for epilepsy control. The patient remained seizure-free for the next 2 years under carbamazepine prescription (400 mg/day). However, the patient experienced further symptoms after antiepileptic therapy was voluntarily terminated 1 month before initial presentation. The timeline since onset of initial symptoms is presented in the Table.

Initial symptoms presented by the patient included focal seizure (left face, left arm, and left leg muscle twitch) and impaired consciousness. After the seizure episode, the patient exhibited poor concentration and frequent fatigue, which rendered her unable to attend school. Four days after the initial symptoms, the patient exhibited behavioral change, agitation, and intermittent focal seizure with left face, left arm and left leg muscle twitch, and she was brought to the emergency room (ER) 2 days later. Focal epilepsy was initially suspected, and an antiepileptic drug was prescribed accordingly. However, due to the persistent symptoms (including frequent focal seizure and agitation), the patient returned to the ER the next day, where orolingual dyskinesia, limb rigidity, bizarre behavior, self-harm behavior, auditory hallucination, and delusion were presented. The patient was later transferred to the intensive care unit (ICU) due to psychosis progression, orolingual dyskinesia, and four-limb rigidity.

Based on the initial clinical presentation, autoimmune encephalitis was suspected. To ensure accurate diagnosis and exclude other potential diagnoses—such as temporal lobe epilepsy with automatism, viral encephalitis, and neuroleptic malignant syndrome—serum tumor markers, autoimmune profiles and autoimmune encephalitis panels, video EEG, brain magnetic resonance imaging (MRI), whole-body computed tomography (CT) scan, and lumbar puncture were arranged and performed.

**Figure 1:**
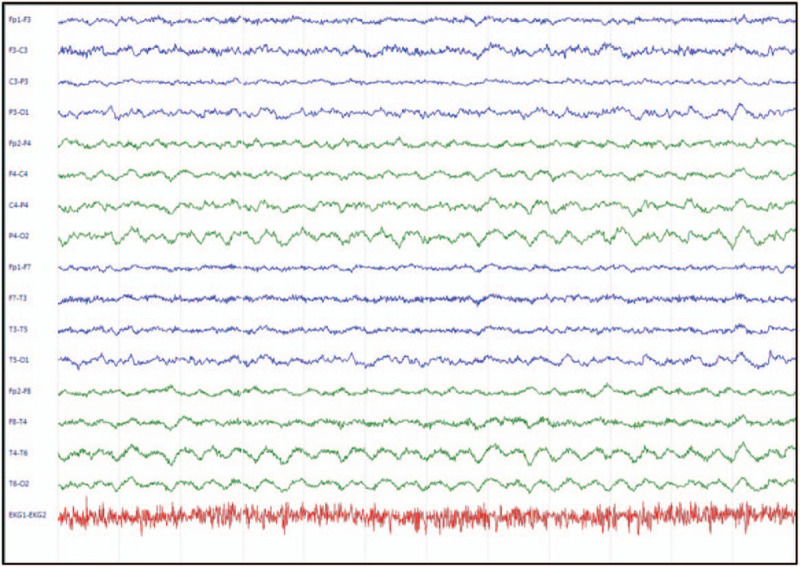
A 24-hour video recorded EEG showing the EDB pattern, prominent fast beta activity overriding slow delta activity in the bifrontal and bitemporal area, in our patient 10 days (D10) after symptom onset. EDB = extreme delta brush, EEG = electroencephalography.

Serum tumor markers and autoimmune profiles yielded negative results. Brain CT and brain MRI results were also negative. Whole-body CT scan showed no apparent tumors. However, video EEG revealed diffuse background slow waves and a suspicious EDB pattern with frontal predominance (Fig. 1). Moreover, serum autoimmune encephalitis panels revealed a modest titer of anti-NMDAR antibody (1:10; Fig. 2). Consistently, lumbar puncture revealed mild pleocytosis, which was compatible with suspected autoimmune encephalitis.

**Figure 2:**
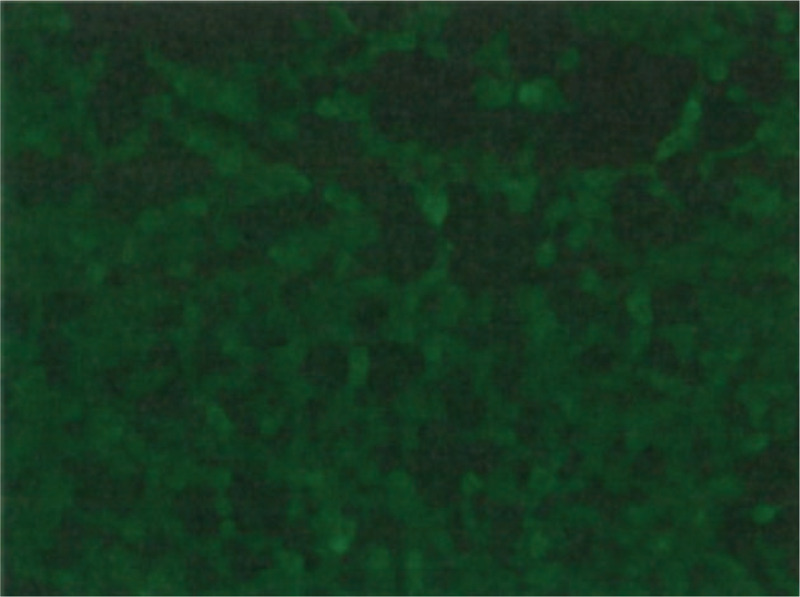
Positive serum immunofluorescence test.

**Figure 3:**
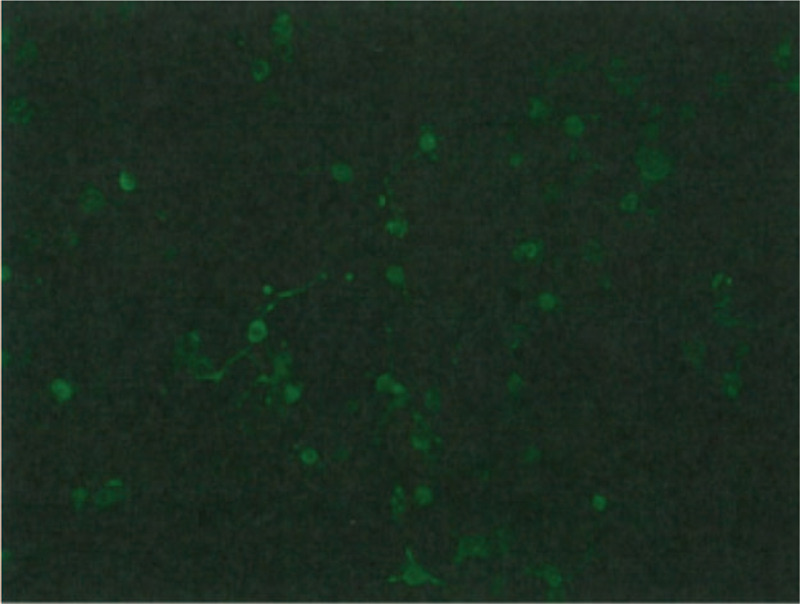
Positive cerebrospinal fluid immunofluorescence test.

Based on the evidence so far, intravenous methylprednisolone (1000 g/day) was prescribed (8 days after symptoms onset, D8). The steroid pulse therapy lasted for 5 days, during which psychosis and four-limb rigidity were ameliorated. However, these episodes became more frequent after treatment was replaced by oral prednisolone (60 mg/day). The dose of antiepileptic drugs was titrated, and other antiepileptic drugs were added according to the progression of motor phenomenon. Video EEG was again arranged to differentiate focal seizure and anti-NMDAR encephalitis-related movement disorder. The results revealed diffuse background slow waves and an EDB pattern, which was compatible with suspected anti-NMDAR encephalitis. Due to absence of epileptiform discharges on video EEG, induction of coma with continuous intravenous anesthetic agents were avoided. However, psychosis and limb rigidity still fluctuated. Finally, 17 days after symptoms onset (D17), the cerebrospinal fluid autoimmune encephalitis panels were positive for anti-NMDAR antibody (Fig. 3), and a diagnosis of anti-NMDAR encephalitis was thus confirmed. On the basis of the diagnosis, clinical recovery status, and age, the patient received second-line immunosuppression therapy (intravenous immunoglobulin, IVIG) the following day (D18).

Remarkably, psychosis and four-limb rigidity became substantially less frequent on the second day of IVIG administration (D19). Moreover, the clinical status of the patient greatly improved in the following 5 days. In particular, the patient exhibited a clear consciousness and good orientation (D23). Given only residual mild cognitive impairment was detected after the Mini-Mental State Examination (MMSE) and an interview, the patient was transferred to the ordinary ward 27 days after symptoms onset (see Table 1).

**Table 1:**
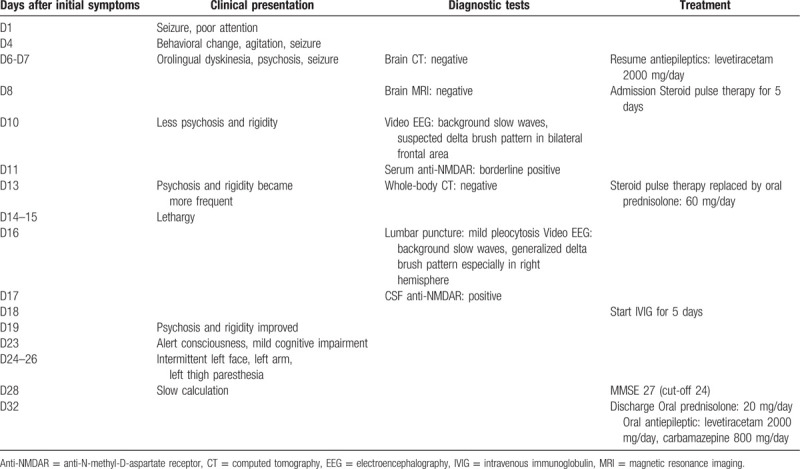
Timeline.

In the ordinary ward, the patient continued to exhibit improvement in cognitive functions, including attention and calculation. The patient also recovered muscle strength. Further awake EEG analysis showed normal background waves with no epileptiform discharges. Although occasional left face, left arm, and left thigh paresthesia were reported, the frequency of paresthesia decreased gradually. Finally, the patient was discharged 32 days after symptoms onset (D32) fully capable of independent activities of daily living.

The early use of steroid pulse therapy achieved clinical improvements during the acute stage, but it did not completely prevent anti-NMDAR encephalitis-related brain damage and subsequent cognitive functional decline (as revealed by the formal MMSE test). However, these were resolved 2 weeks post-discharge, and normal cognitive function was later recovered.

## 3 Discussion

Anti-NMDAR encephalitis is an autoimmune encephalitis that was first described in 2008.^[1]^ It often occurs in children and young adults (a mean age of 21 years) and is more predominant in females.^[5]^ Although it can occur in patients with no tumor growth, NMDAR encephalitis is most frequently associated with ovary teratoma.^[1]^ The clinical picture varies among individuals. Most patients initially experience minor illness or flu-like symptoms. Psychotic symptoms, movement disorder, and seizure may predominate within weeks to months after onset, followed by autonomic dysfunction and death if the disease is allowed to progress. Diagnosis is confirmed by detection of anti-NMDAR antibodies in the serum or cerebrospinal fluid of the patient. Predictors of favorable outcome include early treatment and lack of ICU admission.^[5]^ Treatment includes tumor excision (if present); first-line immunosuppression such as steroid pulse therapy, IVIG and plasma exchange; and second-line immunosuppression such as cyclophosphamide and rituximab. Clinically, appropriate treatment can generally produce good prognostic outcomes.^[6]^

In our case, the clinical picture was made additionally complex due to the concomitant suspected focal seizure attack, previous epilepsy history, and anti-NMDAR encephalitis movement disorder. As intermittent rigidity of all four-limbs and impaired consciousness can be attributed to focal seizure as well as anti-NMDR encephalitis movement disorder, we had to quickly decide whether the patient was experiencing focal seizure with impending status epilepticus or movement disorder due to anti-NMDR encephalitis. We addressed this using 24-hour video EEG, which revealed predominantly diffuse slow background waves and minimal suspected epileptiform discharges. These did not correlate with most clinical motor phenomena. Notably, we observed a prominent EDB pattern in the bilateral frontal area during EEG recording (Fig. 1), which was almost pathognomonic for anti-NMDAR encephalitis. According to a previous study,^[3]^ the EDB pattern is present in up to 30% of anti-NMDAR encephalitis patients. In our case, the presence of anti-NMDAR antibody in the serum of the patient further indicated anti-NMDAR encephalitis.

As mentioned previously, current treatment for anti-NMDAR encephalitis involves first-line immunotherapy and corticosteroid therapy, immunoglobulin and plasma exchange, and tumor excision (if present). There are no significant clinical differences among the 3 first-line immunotherapies when used alone, or among any combinations of 2 of these immunotherapies.^[6]^ With regards to the use of corticosteroid and IVIG, there are no significant differences between the outcomes of early combined treatment and sequential treatment, or between using corticosteroid first and IVIG first.^[6]^ In our case, steroid pulse therapy was immediately prescribed after early recognition of the clinical characteristics and EDB pattern in the patient. This led to a reduction, but not elimination, of the intermittent confusion and psychosis episodes. Subsequently, we introduced IVIG for better immunosuppression. Remarkably, the clinical response was excellent after IVIG treatment, and the patient exhibited clear consciousness and mild cognitive impairment upon discharge. During follow-up at our neurology clinic, the cognitive sequelae of the patient continued to improve and were completely resolved 1 month post-discharge.

Here, we presented a case of subacute onset of focal seizure with psychosis due to anti-NMDAR encephalitis. We demonstrated that psychosis status may be resolved by immunosuppression therapy. The notable points in our case are as follows:

1. the manifestation of anti-NMDAR encephalitis in a patient with previous epilepsy history may increase the complexity and confuse diagnosis at initial clinical presentation;
2. the typical EDB pattern may guide early diagnosis of anti-NMDAR encephalitis; and
3. early and aggressive immunotherapy can induce excellent clinical response with only mild cognitive sequelae in patients with anti-NMDAR encephalitis.

Thus, we conclude that the early recognition and diagnosis of anti-NMDAR encephalitis are crucial for favorable clinical outcomes. We further propose that anti-NMDAR encephalitis should always be considered when subacute focal seizure, psychosis, and compatible EEG findings (i.e., an EDB pattern) are present in patients.

## Author contributions

**Writing – original draft:** Yu-Ming Chen.

**Writing – review & editing:** Yu-Ming Chen, Pei-Hsin Kuo.
